# Importance of Bile Composition for Diagnosis of Biliary Obstructions

**DOI:** 10.3390/molecules26237279

**Published:** 2021-11-30

**Authors:** Łukasz Krupa, Robert Staroń, Dorota Dulko, Natalia Łozińska, Alan R. Mackie, Neil M. Rigby, Adam Macierzanka, Aleksandra Markiewicz, Christian Jungnickel

**Affiliations:** 1 Teaching Hospital No 1, Department of Gastroenterology and Hepatology with Internal Disease Unit, Chopina 2, 35-055 Rzeszów, Poland; krupasl@yahoo.com (Ł.K.); rob.staron@gmail.com (R.S.); 2 Medical Department, University of Rzeszów, Kopisto 2a, 35-310 Rzeszów, Poland; 3 Department of Colloid and Lipid Science, Faculty of Chemistry, Gdańsk University of Technology, Narutowicza 11/12, 80-233 Gdańsk, Poland; dorota.dulko@pg.edu.pl (D.D.); s154531@student.pg.edu.pl (N.Ł.); adamacie@pg.edu.pl (A.M.); 4 School of Food Science & Nutrition, University of Leeds, Leeds LS2 9JT, UK; A.R.Mackie@leeds.ac.uk (A.R.M.); N.M.Rigby@leeds.ac.uk (N.M.R.); 5 Laboratory of Translational Oncology Intercollegiate, Faculty of Biotechnology, University of Gdańsk and Medical University of Gdańsk, 80-211 Gdańsk, Poland; aleksandra.markiewicz@biotech.ug.edu.pl

**Keywords:** bilirubin, conjugated/unconjugated bile salts, biliary obstruction, pancreatic neoplasm, cholangiocarcinoma, choledocholithiasis

## Abstract

Determination of the cause of a biliary obstruction is often inconclusive from serum analysis alone without further clinical tests. To this end, serum markers as well as the composition of bile of 74 patients with biliary obstructions were determined to improve the diagnoses. The samples were collected from the patients during an endoscopic retrograde cholangiopancreatography (ERCP). The concentration of eight bile salts, specifically sodium cholate, sodium glycocholate, sodium taurocholate, sodium glycodeoxycholate, sodium chenodeoxycholate, sodium glycochenodeoxycholate, sodium taurodeoxycholate, and sodium taurochenodeoxycholate as well as bile cholesterol were determined by HPLC-MS. Serum alanine aminotransferase (ALT), aspartate transaminase (AST), and bilirubin were measured before the ERCP. The aim was to determine a diagnostic factor and gain insights into the influence of serum bilirubin as well as bile salts on diseases. Ratios of conjugated/unconjugated, primary/secondary, and taurine/glycine conjugated bile salts were determined to facilitate the comparison to literature data. Receiver operating characteristic (ROC) curves were determined, and the cut-off values were calculated by determining the point closest to (0,1). It was found that serum bilirubin was a good indicator of the type of biliary obstruction; it was able to differentiate between benign obstructions such as choledocholithiasis (at the concentration of >11 µmol/L) and malignant changes such as pancreatic neoplasms or cholangiocarcinoma (at the concentration of >59 µmol/L). In addition, it was shown that conjugated/unconjugated bile salts confirm the presence of an obstruction. With lower levels of conjugated/unconjugated bile salts the possibility for inflammation and, thus, neoplasms increase.

## 1. Introduction

Biliary obstruction refers to a blockage of any duct that carries bile from the liver and from the gallbladder to the small intestine. This can occur at any of the levels of the biliary system. The symptoms of biliary obstruction result from the accumulation of bilirubin in the blood. Biliary obstructions may be caused by benign or malignant diseases of the alimentary tract. The main cause of benign biliary obstruction is choledocholithiasis due to gallstone formations [1]. Other benign causes include strictures post cholecystectomy, inflammatory stricture formation secondary to cholangitis, pancreatitis, and idiopathic causes. Other rarer causes of nonmalignant obstructions are choledochal cysts, primary sclerosing cholangitis, and Mirrizi syndrome [2]. In addition, liver transplantation may cause biliary track dysfunction in 13–35% of patients [3].

The malignant process also promotes the formation of biliary strictures [4]. Malignant obstructions are most commonly caused by cholangiocarcinoma and pancreatic cancer. Other causes are gallbladder cancer, compression by malignant lymph nodes, and metastasis [5,6]. The differentiation between malignant and benign causes is very important for clinicians as most malignant obstructions, like those caused by cholangiocarcinoma and pancreatic cancer, are unresectable at the time of diagnosis, and treatment options are restricted to palliative management, which typically involves stent insertion as a mainstay of treatment. The survival of patients with malignancies affecting the biliary duct varies between 3–10 months [7]. In cases of pancreatic cancer, the median survival time is 8 months [8]. Inoperable tumors decrease the survival potential further [9].

Types of bile duct strictures are difficult to differentiate by noninvasive methods [10], such as radiological imaging alone. The noninvasive radiological modalities for evaluation of these patients include ultrasonography, contrast-enhanced CT scans, MRI, and magnetic resonance cholangiopancreaticography (MRCP). These noninvasive diagnostic methods may provide information about the level of obstruction, the extent of biliary dilatation, and the presence of a mass or distant metastasis [11,12] and are crucial for further treatment of the patient. On the other hand, endoscopic retrograde cholangiopancreatography (ERCP), percutaneous transhepatic cholangiography (PTC), and endoscopic ultrasound (EUS) are invasive tests, which provide additional imaging information and allow tissue sampling and treatment during the same session. One of the commonly used methods for biliary stricture diagnosis and treatment is ERCP, which provides histopathological tissue diagnosis in 35% of cases and shows a 100% specificity rate for the malignancy diagnosis (pancreatic cancer, biliary cancer, cancer of the ampulla of Vater, metastatic diseases involving bile ducts, and other rare causes) [13]. Techniques providing the tissue for cytological or histological diagnosis include the collection of bile [14], brush cytology [15], or forceps biopsy [16], and direct cholangioscopy.

Despite the advances in imaging modalities and new endoscopic techniques, differentiating between benign and malignant causes of biliary obstructions remains challenging. Endoscopic techniques of tissue acquisition such as biopsies, brushings, and fine needle aspiration (FNA) may provide a definitive tissue diagnosis; however, the combined sensitivity of these techniques is in the region of 60% [17,18]. The sensitivity of endoscopic ultrasound (EUS) and FNA for the diagnosis of a malignant biliary obstruction ranges widely from 43% to 86% [19,20,21]. The combination of ERCP and EUS may improve the rate of histological confirmation of malignancy. All the endoscopic tests are invasive and associated with risks from complications. The initial diagnosis is based on a review of clinical, biochemical, and radiological features.

Although biliary strictures present a diagnostic challenge and are hard to differentiate, the laboratory parameters may help to indicate the types of strictures [22]. The laboratory values of the liver function tests in the serum and current tumor marker levels lack sufficient specificity to determine the precise cause of a biliary obstruction [23,24,25]. Serum bilirubin levels are a strong predictor of biliary malignant diseases, with the optimum sensitivity and specificity for malignancy at bilirubin levels of >100 µmol/L [26]. Patients with cholangiocarcinoma had elevated bilirubin levels (60–470 µmol/L) [27]. Raised bilirubin levels were also associated with the development of pancreatic cancer [28] and the increased risk of gallstone formation [29].

Bile salts (BSs) may play an important role in the determination of the cause of stricture formation. BSs are synthesized from cholesterol in the liver and stored in the gallbladder [30]. Cholic acid and chenodeoxycholic acid are two primary bile acids (BAs) synthesized in the human liver. BAs undergo modification by the liver and are conjugated with the glycine or taurine molecule by BA-CoA amino acid N-acyltransferase (BAAT) to form BSs [31]. This process ensures lowering the pK_a_ value of the formed BSs [32]. Thus, at the physiological pH, the conjugated BSs appear in the ionized form. BSs are transported across the canalicular membrane to the gallbladder, from where they are secreted in the bile to the duodenum after meal intake [33]. In the duodenum and onwards, the formation of deconjugated BSs, deoxycholic acid, and lithocholic acid can occur due to the presence of intestinal bacteria that secrete the bile salt hydrolase (BSH), an enzyme responsible for this conformational change. The secondary BSs may be further conjugated with glycine and taurine molecules to form secondary conjugated BSs. Bile salts are natural ligands for the nuclear BA receptor, Farsenoid X receptor (FXR), and are responsible for the activation of FXR [34]. Activated FXR inhibits the expression of the CYP7A1 enzyme which is responsible for the synthesis of the BSs from cholesterol in the liver [35]. Therefore, BSs are able to control their own synthesis. FXR supresses the CYP7A1 gene expression by induction of the hepatic small heterodimer partner (SHP, NROB2), which inhibits activity of the tissue specific liver receptor homolog 1 (LRH-1), which is responsible for controlling the expression of the CYP7A1 enzyme, and via the induction of the ileal hormone fibroblast growth factor 19 (FGF19) in humans and FGF15 in mice [34,36,37]. Moreover, expression of the hepatic BA transporters is also controlled by FXR. The BA transporters, the Na^+^ taurocholate cotransporting polypeptide (NTCP), the bile salt export pump (BSEP), the apical sodium-dependent BA transporter (ASBT), and the organic solute transporter OSTα-OSTβ are responsible for controlling the absorption rate of BSs, the enterohepatic circulation, and the removal of BSs from the body [38]. FXR is also responsible for regulation of BAAT [39].

BSs are transported down from the liver and through the biliary tree to the gallbladder [40]. After the BSs have contributed to food digestion and nutrient absorption, the majority (>90%) are reabsorbed by active transport at the terminal ileum [40] to the liver in a process known as enterohepatic circulation [41]. This signifies that BSs can be obtained from either de novo synthesis or can be recycled from enterohepatic circulation. The transport of the BSs from the blood to the hepatocytes takes place with assistance from sodium-dependent cotransporters [42].

It has been known since 1939 that BSs act as potential carcinogens [43] with a cytotoxic effect on hepatocytes and enterocytes [44] and negatively affect the mucosa of the stomach, intestine, and gallbladder [45]. Specifically, secondary unconjugated BSs, due to their higher hydrophobicity, are more toxic than their primary forms [44]. They promote oxidative/nitrosative stress, cause DNA damage, and promote apoptosis and mutation [46]. Conversely, Dai et al. [47] have indicated that unconjugated BAs promote cell apoptosis and reduce the growth of cholangiocarcinoma cells, whereas conjugated BSs promote cell growth. This would indicate that the exact nature of the role of BAs in cancer formation is as yet poorly understood. However, it is clear that an interaction exists, as it has been shown that the concentration of conjugated BSs in benign biliary diseases has been shown to be statistically lower than in cholangiocarcinoma patients [48]. Zhang et al. [49], as one of the only reports on BAs in serum, have proposed BAs as the biomarkers for cholangiocarcinoma since the ratio of conjugated to unconjugated BAs in cholangiocarcinoma patients was shown to be reduced. Therefore, the imbalance of the BA composition was indicated to play a crucial role in the development of cholangiocarcinoma [50]. Transporters located in the canalicular membrane allow for the secretion of BSs from hepatocytes. Inhibition of the BS secretion results in the pathophysiologic concentration of BSs named cholestasis. How these cause cancer has been postulated through investigation using cultured rat hepatocytes [51]. Jaeschke et al. postulated that elevated BS concentrations cause the translocation of the intracellular Fas ligand (can induce apoptosis and is a tumor necrosis factor) to the cell membrane and trigger cell death [51]. Lower in the biliary tree, BAs have been suggested to be a key factor influencing the development of pancreatic cancer [52,53,54]. Rees et al. [55] have compared the level of BAs in patients with pancreatic cancer and with benign disease. Increased concentrations of the unconjugated BAs in the malignant group may be explained by the bacteria proliferation in and around the common bile duct.

Therefore, an alteration in the conjugation level and an obstruction-induced reduction of BS concentration in the small intestine may change the BS synthesis via action of FXR and promote excessive BA synthesis. Imbalance in the BA composition can be caused by a number of factors, e.g., 1. altered synthesis due to FXR promotion or inhibition [47,56]; 2. change in the function of BAAT; 3. change in the function of the BA transporters [57]; 4. change in the external concentration of the BS due to a blockage [58,59,60]; 5. changed function of BSH [58,59,60].

Therefore, the aim of this study was to analyze the significance of serum bilirubin as an indicator for biliary obstructions as well as to analyze the causal and diagnostic significance of bile salts in biliary obstructions. To achieve this aim, we have collected and analyzed human bile from 150 patients diagnosed with various biliary obstructions occurring at different levels of the biliary tree.

## 2. Results and Discussion

### 2.1. Bilirubin as an Indicator of Neoplasm

**Figure 1:**
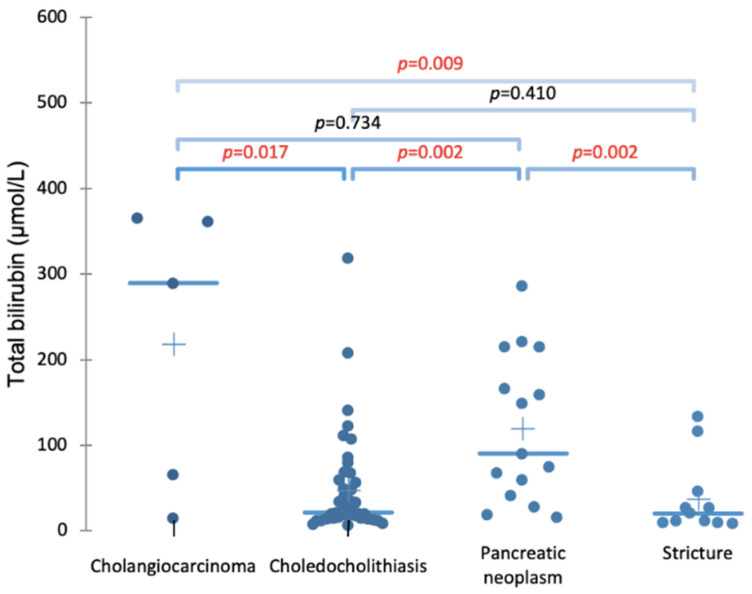
Comparison of the bilirubin levels in the serum of patients with different conditions resulting in biliary blockage. It can clearly be seen that the bilirubin levels in the case of a neoplasm (either cholangiocarcinoma or pancreatic) is significantly higher compared to benign strictures or choledocholithiasis. *p*-values were determined using the Kruskal-Wallis test with the Dunn post hoc nonparametic comparison.

The importance of bilirubin levels in diseases is shown in Figure 1, which clearly differentiates neoplasms (cholangiocarcinoma and pancreatic cancer) from the nonmalignant diseases (choledocholithiasis and stricture).

### 2.2. Importance of BSs as Indicators of Biliary Obstruction

**Figure 2:**
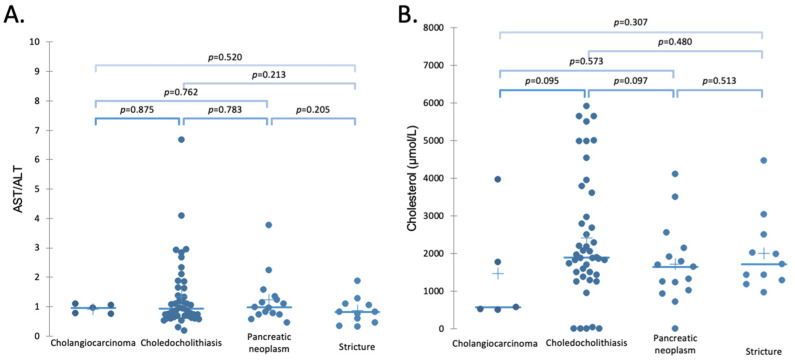
AST/ALT ratio (**A**) and cholesterol levels (**B**) measured in bile collected from patients with different malignancies. (**A**). Traditional liver diagnostic tests. AST/ALT ratio were not able to successfully differentiate the biliary blockages. (**B**). Cholesterol levels of the bile were not significantly different in any of the disease groups; however, the highest cholesterol levels were found for the choledocholithiasis patients.

Table 1 shows the average levels of measured compounds with the standard deviation in each of the four classified pathologies. The ratios are calculated as a relative ratio, relative to the number of items. The concentrations of different BSs measured in the bile and blood of 63 patients with pathological biliary obstructions were compared to 11 patients with benign strictures (postinflammatory, postsurgical, iatrogenic, idiopathic), since it is not ethical to extract bile from healthy patients. However, literature values for healthy patients are shown indicatively. Figure 2 shows the cholesterol and liver functions of the recorded patients.

#### 2.2.1. Choledocholithiasis

**Figure 3:**
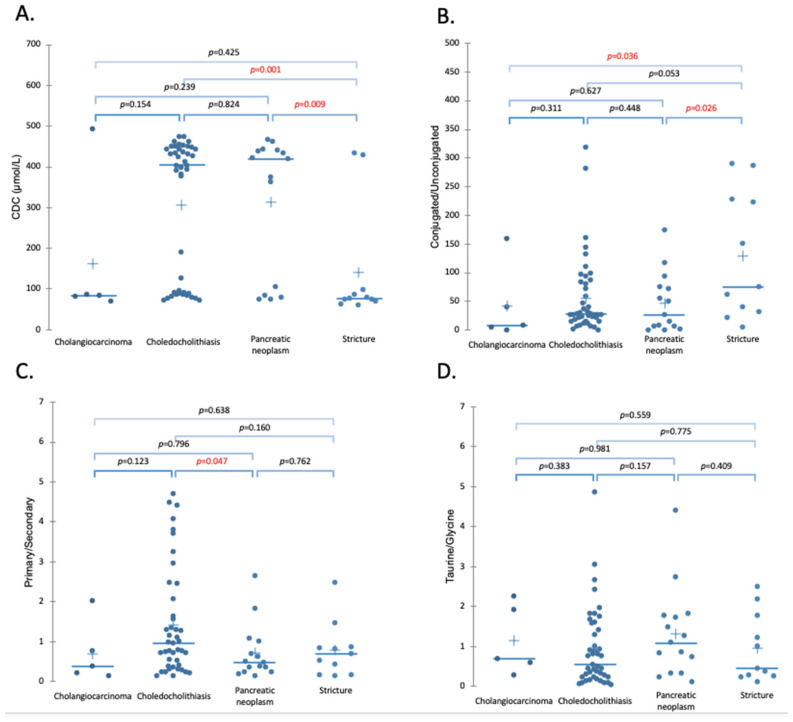
(**A**). Changes of CDC with respect to disease type. The statistical difference between strictures and pancreatic neoplasms as well as choledocholithiasis was indicated. Concentrations of CDC are altered with the development of gallstones and pancreatic cancer. (**B)**. Conjugated/unconjugated ratios of BSs in patients with different malignancies. Elevated ratios indicate increasing conjugated forms of the BSs and simultaneous depletion of unconjugated forms. (**C**). Primary/secondary ratios of BSs. The ratios were most influenced by the presence of biliary stones. (**D**). Taurine/glycine ratios do not show any changes with disease types.

Our results have shown that BS concentration may function as the potential indicators of choledocholithiasis (when a gallstone blocks the bile duct). The patients with diagnosed choledocholithiasis have shown elevated primary/secondary (P/S) ratios compared to pancreatic neoplasms and elevated conjugated/unconjugated (C/U) ratios of BSs when compared to benign strictures as shown in Figure 3B,C. Blockage resulting from the gallstone formation inhibits the flow of the BSs from the gallbladder to the small intestine leading to dysbiosis and, therefore, a reduction in BSH [58,59,60]. Depletion of BSH leads to suppression of the deconjugation process. Conjugated BSs are better ligands for FXR than their unconjugated forms [56]. Therefore, alterations in the conjugation levels and reduced concentrations in the small intestine due to obstructions may change BS synthesis via action of FXR and promote excessive BA synthesis. Those alterations of the expression of the FXR result in the increased expression of CYP7A1, which promotes the excessive synthesis of the BSs and consequently leads to the changed composition of the BA pool size [76]. Those alterations of BA synthesis result in a predominant concentration of primary BSs in patients with cholestasis [59], as can be observed in Figure 3C. The accumulated primary synthesized BSs may be excessively conjugated by the BA-CoA:amino acid N-acyltransferase (BAAT) enzyme, which is reflected by the elevated C/U ratio observed in Figure 3B. Disorders caused by the reduced flow of BSs and excessive BA synthesis lead to the formation of conjugation forms of BSs, which then may be responsible for the development of cholangiocarcinoma [47]. Interestingly, the cholesterol in the bile was not found to be significantly elevated compared to the other diseases (Figure 2B). The cholesterol level was shown to be correlated with the level of the C/U ratio of BS (Pearson test, *p* < 0.001, r = 0.303). Therefore, excessive levels of the chenodeoxycholic acid (Figure 3A), responsible for the solubilization of cholesterol, may explain the insignificant elevation of cholesterol levels.

#### 2.2.2. Cholangiocarcinoma

Strom et al. [77] noticed the differences in the BA compositions among control groups, subjects with gallstones, and subjects with bile duct cancer. Depletion of deoxycholic and lithocholic acids was noticed for biliary tract cancer [66]. The reduction in the secondary unconjugated BS level was due to the limited flow of the primary BSs to the small intestine. Hence, the decreased level of reabsorbed BSs increase activation of CYP7A1. Increased BS synthesis results in elevated levels of primary over secondary BSs, which can be observed in Figure 3C. Moreover, after reabsorption to the liver, the secondary unconjugated BSs may be conjugated with taurine or glycine, which are catalyzed by BAAT. Reduced flow of the BSs and their excessive accumulation not only change the action of the BS enzymes but also promote the creation of elevated conjugated forms of the BSs, which is reflected in the elevated C/U ratio (Figure 3B). Disorders caused by reduced flow of the BSs and excessive BA synthesis lead to the formation of elevated conjugation forms, which may be responsible for the development of cholangiocarcinoma [47]. Increased ratios of C/U BSs in patients with cholangiocarcinoma were also observed by Zhang et al. [49].

#### 2.2.3. Pancreatic Neoplasms

Increased production of secondary BSs may be correlated with the induction of tumors at the head of the pancreas, which leads to bile duct obstruction as was suggested by Rees et al. [55]. Moreover, elevated secondary BS levels promote the generation of reactive oxygen species and induce DNA damage and cell disruption.

**Figure 4:**
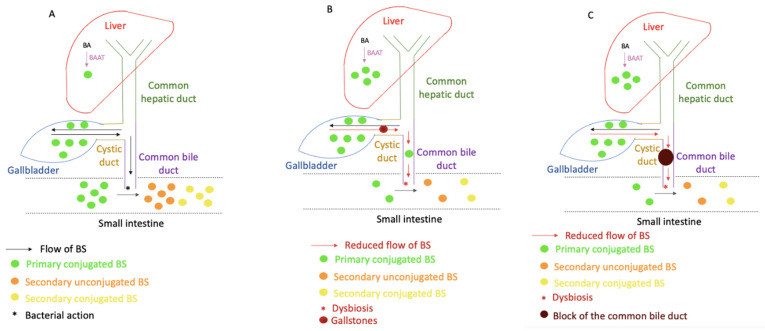
(**A**). The normal flow of the BSs from the liver, through the hepatic duct, cystic duct, gallbladder, and common bile duct to the small intestine. BSs are retransported back to the liver after fulfilling their role. They control their own synthesis by inducing expression of FXR. (**B**). As a result of gallstone formation, the flow of BSs is reduced. (**C**). Blockage of the BS flow caused by the development of cholangiocarcinoma. Decreased levels of reabsorbed BSs reduce the FXR signalling and promote activation of CYP7A1 enzyme leading to increased BA synthesis.

Pancreatic neoplasms have shown different P/S ratios than choledocholithiasis, as can be observed in Figure 3C. Decreased concentrations of secondary BSs can be a result of reduced flow of the primary BSs, caused by the obstruction. Therefore, lower amounts of the primary BSs can be transported to the small intestine and undergo the deconjugation process. The reabsorbed BSs will be further conjugated in the liver to the secondary conjugated (SC) BSs. Moreover, lower concentrations of absorbed BSs may result in the higher activation of the CYP7A1 enzyme and increased BA synthesis as well as the increased production of more primary BSs. Therefore, previously accumulated and newly synthesized BSs may undergo the conjugation process, which may result in excessive conjugation levels, as reflected in Figure 4B by the elevated C/U ratios. The P/S ratios were shown to be statistically different between cholangiocarcinoma and pancreatic neoplasms (Figure 4). The differences could result from the location of the cancer and more efficient blockage of the BS flows.

The schematic representation of possible changes in BS composition due to the development of diseases is shown in Figure 4. Under normal conditions, the BAs are synthesized in the liver and transported to the gallbladder (Figure 4A). They are released to the small intestine during the consumption of a meal, and after fulfilling their roles they are reabsorbed by the liver, and induce the expression of FXR, which inhibits activation of CYP7A1 and regulates BA synthesis. Strictures created during the benign state of disease lead to accumulation of the synthesized BSs and reduce the flow of the BSs to the small intestine (Figure 4B). The gallstones formed in the gallbladder may accumulate and form bigger aggregates in the common bile duct, which lead to decreased BS flow to the small intestine.

As can be seen in Figure 2A, patients with biliary obstructions and completely normal liver function tests (LFTs) are unlikely to have malignant pathologies, where only choledocholithiasis shows elevated AST/ALT ratios, albeit without statistical significance [78].

**Figure 5:**
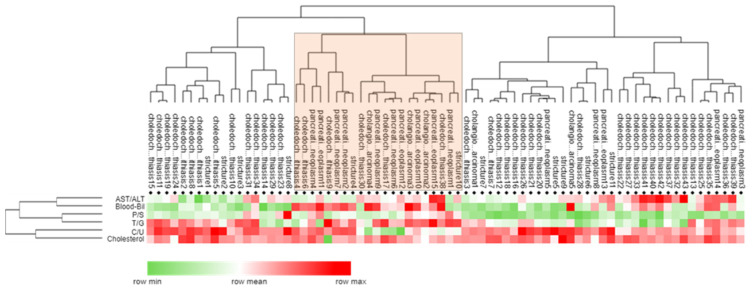
Heatmap and dendrogram of the diagnostic ratios (AST/ALT, conjugated/unconjugated, primary/secondary, taurine/glycine conjugated bile salts), biliary cholesterol, and serum bilirubin in the tested patients with different malignancies. Presence of pancreatic cancer-enriched samples can be identified in the cluster marked with orange (10/15 of pancreatic cancers occur in this cluster).

When analyzing the heatmap (Figure 5), BA ratios were visibly altered for choledocholithiasis compared to the other pathologies.

### 2.3. Diagnosis of Biliary Obstruction

**Figure 6:**
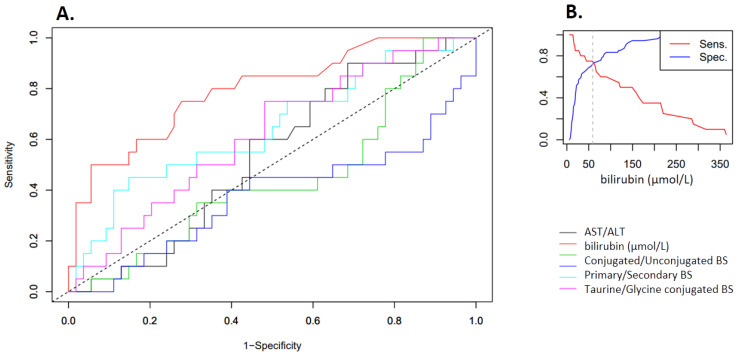
(**A**). ROC curves for the determination of diagnostic values of various markers to discriminate neoplasms (pancreatic cancer or cholangiocarcinoma) vs. nonmalignant changes (stricture and choledocholithiasis). Only bilirubin (AUC 0.793 with a *p* < 0.001). was statistically significant (**B**). Sensitivity and Specificity curve for bilirubin concentration clearly indicates a specificity of close to 77% if serum level is over 55 µmol/L.

When looking at the diagnostic significance of individual markers in receiver operating characteristic (ROC) curves (Figure 6), only serum bilirubin shows an appreciable diagnostic relevance, with AUC = 0.793, *p* < 0.001. None of the other values showed the same significance. However, the results indicate that there is a correlative relationship between the BS composition (as represented by the C/U ratio) and the various diseases.

**Figure 7:**
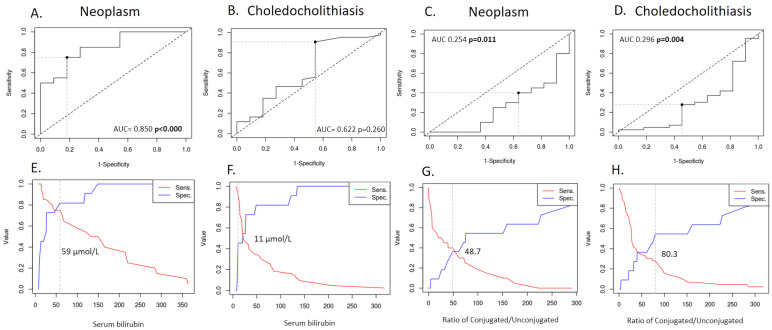
(**A**–**D**) ROC curves comparing neoplasms (both cholangiocarcinoma and pancreatic, (**A**,**C**)) and choledocholithiasis cases (**B**,**D**) to the benign stricture cases using two indicators: serum bilirubin and the ratio of conjugated/unconjugated BSs. In three cases (*p*-value marked in bold), the ROC curve presented a statistically significant indicator. The corresponding cut-off values (**E**–**H**) are given under each ROC curve.

Figure 7 clearly indicates the importance of bilirubin as an indicator of malignant biliary obstruction, where the cut-off value for bilirubin concentration serves as an indicator for the type of disease (i.e., above 11 µmol/L choledocholithiasis is likely, and above 59 µmol/L a neoplasm may be suspected). This is in line with the previous model of the less complete closure caused by the cholestasis [79]. The ratio of conjugated to unconjugated BSs was found to be a significant indicator for healthy patients (thus the line is below the diagonal [80]) when compared with cholestasis and neoplasm patients, with a ratio of 80.3 and 48.7, respectively. It indicates that neoplasms are characterized by almost a 50% reduction in the amount of conjugated BSs in the biliary tract (as shown in Figure 3B average C/U changes from ~50 for malignant strictures to ~120 for benign strictures). This could be due to the lower hepatic recycling of the BSs since all the conjugated as well as unconjugated BSs measured derived from a primary synthesis. The only unconjugated BAs included in our test set was a primary bile salt (sodium chenodeoxycholate). Therefore, the stronger the stricture the less bile can recirculate. This can also be seen when grouping bilirubin levels into three categories (as a result of the ROC analysis), low (≤110 µmol/L), medium, and high (≥59 µmol/L), and grouping these (using the Kruskal Wallis test with a Dunn post hoc test) with the three bile salt ratios (C/U, P/S, and T/G). The only correlation was found for C/U at low and medium bilirubin levels (with *p* values < 0.001, the *p* values for P/S, T/G and cholesterol where 0.242, 0.192, and 0.647 respectively).

This would indicate that the presence of unconjugated BSs increase the risk of a pathological indication. This is in line with the previous effect of unconjugated BSs on tissue (i.e., cytotoxicity and inflammation). The presence of unconjugated BSs present a higher cellular stress and may result in an inflammatory response. In fact, all biliary diseases are related to rates of inflammation in biliary tissue [81,82]. This indicates that blockage of the biliary tract can lead to self-propagating inflammation, resulting from the increased synthesis of BSs, and a loss of function of BA-CoA:amino acid n-acyltransferase (BAAT). The loss of BAAT is thought to be due to 4-hydroxynonenal (4HNE) in a dose-dependent relationship [83]. 4HNE is a well-studied aldehyde that has been shown to be directly related to oxidative stress [84].

## 3. Materials and Methods

### 3.1. Sample Collection

The collection and study of human bile (HB) samples were approved by the ethics committee of the Regional Medical Chamber in Rzeszów, Poland (certificate 15/B/2016). All methods were planned and conducted in accordance with the ethical principles outlined in the Declaration of Helsinki.

Samples of HB were collected at the Department of Gastroenterology and Hepatology (Teaching Hospital No. 1, Rzeszów, Poland) during an endoscopic retrograde cholangio-pancreatography (ERCP). ERCP is an endoscopic procedure which involves the assessment and therapy of the bile ducts and/or pancreatic ducts.

For the purpose of our study, we included patients for whom therapeutic procedures on bile ducts were indicated. After insertion of a duodenoscope into the second part of the duodenum, the ampulla of Vater was identified. The ampulla is located at the major duodenal papilla. All patients who undergo the ERCP procedure must have evidence of biliary or pancreatic duct obstruction. This was confirmed by imaging tests such as: transabdominal ultrasound (USS, computed tomography, magnetic resonance imaging (MRI), or endoscopic ultrasound (EUS)). We recruited 150 patients who were qualified for the ERCP procedure due to imaging evidence of biliary obstruction over the period of 4 months. In order to minimize the risk of complications during the procedure, we decided that a bile aspiration attempt could not take longer than 60 s. In the case of difficult procedures such as the need for a contrast injection before cannulation of the bile duct or the need for a precut or prolonged aspiration attempt, the procedure was completed without bile aspiration. Out of 150 patients recruited for the study, bile from 74 subjects was collected.

As a routine part of ERCP, the ampulla is selectively cannulated with a dedicated sterile catheter, which was inserted selectively over the guide-wire into the bile duct. The position of the catheter was confirmed under fluoroscopy (X-ray guidance).

A syringe was attached to one end of the catheter. The assisting endoscopy nurse performed the aspiration of the bile by applying a gentle suction with a syringe. The catheter was moved back and forward from the extrahepatic bile ducts to the intrahepatic bile ducts. Approximately 2–3 mL of fluid was aspirated. Immediately after aspiration, the samples were sealed and instantly immersed in liquid nitrogen for snap freezing. Samples were stored at −80 °C prior to further examination. The ERCP procedure was completed as planned according to indications.

Serum alanine aminotransferase (ALT), aspartate transaminase (AST), and bilirubin were measured before the ERCP procedure.

### 3.2. HB Analysis

**Figure 8:**
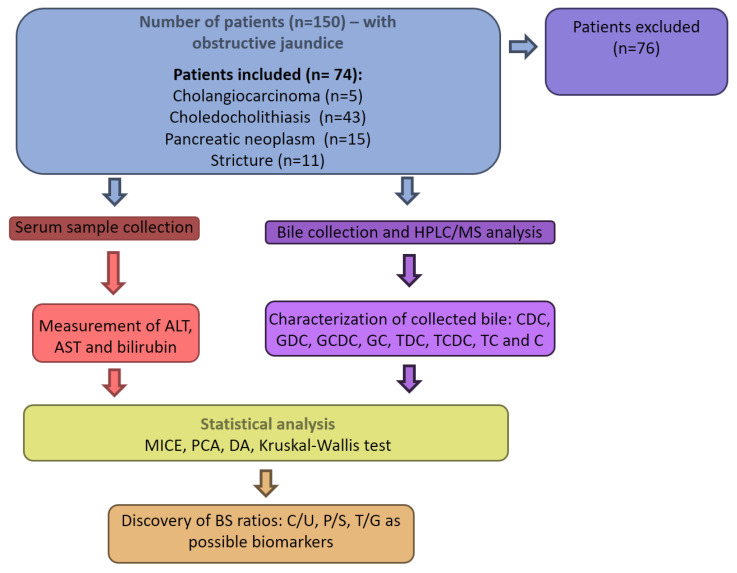
Flowchart from sample collection to analysis and statistical evaluation.

The BS compositions of all HB samples were analyzed using an Agilent 1260 HPLC system coupled to an AB Sciex 4000 QTrap triple quadrupole MS (Sciex, Cheshire, UK). An aliquot of HB (10 µL) was diluted with 0.9% NaCl (990 µL). Diluted HB (50 µL) was transferred into a HPLC vial and mixed with 50 µL methanol. The MS analysis was carried out according to the method described previously [85]. The following BS reference standards were used: sodium cholate (C; C6445, Sigma-Aldrich, Dorset, UK), sodium glycocholate (GC; G7132, Sigma-Aldrich), sodium taurocholate (TC; 86339, Sigma-Aldrich), sodium glycodeoxycholate (GDC; G9910, Sigma-Aldrich), sodium chenodeoxycholate (CDC; C8621, Sigma-Aldrich), sodium glycochenodeoxycholate (GCDC; G0759, Sigma-Aldrich), sodium taurodeoxycholate (TDC; T0875, Sigma-Aldrich), and sodium taurochenodeoxycholate (TCDC; T6260, Sigma-Aldrich). The workflow is shown in Figure 8.

### 3.3. Statistical Analysis

From the 74 patients, 6 patients had incomplete ALT, AST, and serum bilirubin data. The missing data was imputed using MICE with five imputations. The exact procedure for MICE has been described by Łozińska et al [85].

The Kruskal Wallis test with the Dunn post hoc analysis for comparison of continuous variables in multiple groups was used, using XLSTAT (version 2020.1.3.65326). The heatmap and dendrogram were created using GenePattern Version v3.9.11-rc.5 b234 [86]. Raw data were log2-transformed, Pearson correlation was used as a distance measure in columns and row clustering. Data were row-centered by subtracting the row mean from all the values in each row. A summary of the measured data is provided in Table 1. Receiver operating curves (ROC) [87] were generated using the easyROC (v1.3.1), where the cut-off criteria was determined by minimizing the distance to the corner 0,1. 59 µmol/L. Ratios of conjugated/unconjugated (C/U), primary/secondary (P/S), and taurine/glycine conjugated (T/G) BSs were calculated using the average values of both the denominator and numerator.

## 4. Conclusions

After analyzing serum and bile markers in 74 patients presenting a biliary or pancreatic obstruction, we show that the level of serum bilirubin can be used as an initial simple, noninvasive screening test for predicting whether the obstruction is likely to be malignant or benign. In addition, we have analyzed the BS composition. Even though BSs play an important role in disease initiation and progression, the changes in composition are not specific enough to serve as markers.

Monitoring changes in the bile composition might allow for possible novel treatment strategies of the disease. For example, patients with cholangiocarcinoma were shown to exhibit significant imbalances in the ratios of conjugated to unconjugated BSs. This might be partially due to the (self-stimulated) excessive BA synthesis, promoted by the reduction of bile flow and the increased activation of the CYP7A1 enzyme, resulting in an elevated level of conjugated BSs. Inhibiting BS synthesis with FXR antagonists such as guggulsterone [47] may present a promising method to reduce inflammation and thereby inhibit the self-propagating disease development.

With the development of diseases, the BSs undergo specific changes. It is important, therefore, to follow the concentration changes for further development of new markers of the diseases. We recognize the limited cohort size of this study; however, the indications that disruption of BS homeostasis leads to the development of cholangiocarcinoma is a clear conclusion from our work. Furthermore, detailed studies, including high throughput metabolomic profiling are therefore required. Even though the lack of healthy controls is a clear issue with the analysis, obtaining aspirated bile from healthy patients raises ethical questions.

## Figures and Tables

**Table 1:** Summary of the data from the 74 cases of biliary obstruction. Shown are the average levels of measured compounds with the standard deviation in each of the four classified pathologies. The ratios are calculated as a relative ratio, relative to the number of items. When comparing literature values of serum and bile levels, it was shown that the ratios of conjugated/unconjugated, primary/secondary, and taurine/glycine conjugated BSs did not differ between the serum of healthy patients and the collected bile of patients (with *p* values of 0.221, 0.053, and 0.355, respectively).

	Cholangiocarcinoma	Choledocholithiasis	Pancreatic Neoplasm	Stricture
Number of patients	5	43	15	11
Age (years)	72.6 ± 13.74	67.14 ± 17.96	71.00 ± 14.40	70.36 ±14.94
BS concentrations:				
Chenodeoxycholic acid (mmol/L)	0.16 ± 0.18	0.31 ± 0.17	0.31 ± 0.17	0.14 ± 0.14
Glycodeoxycholic acid (mmol/L)	5.00 ± 7.03	5.36 ± 8.53	2.02 ± 3.78	7.31 ± 13.23
Glycochenodeoxycholic acid (mmol/L)	11.17 ± 16.02	20.70 ± 17.57	26.81 ± 50.63	25.25 ± 24.28
Glychocholic acid (mmol/L)	6.41 ± 9.98	9.54 ± 6.71	10.46 ± 13.09	15.49 ± 10.91
Taurodeoxycholic acid (mmol/L)	4.11 ± 5.63	2.61 ± 3.72	1.12 ± 1.66	2.18 ± 3.11
Taurochenodeoxycholic acid (mmol/L)	4.80 ± 6.56	7.30 ± 6.77	6.50 ± 5.63	6.20 ± 4.10
Taurocholic acid (mmol/L)	6.25 ± 9.17	9.73 ± 9.36	9.46 ± 8.87	9.27 ± 5.97
Calculated ratios from literature:				
ALT/AST				
Conjugated/Unconjugated BS	112.56–158.14 [61,62]	2.14–156.85 [62,63,64,65]	19.2–43.57 [55,62]	
Primary/Secondary BS	14.27–62.69 [61,62,66]	2.91–17.71 [59,62,63,64,66,67,68,69,70,71]	2.79–13.17 [55,62]	
Taurine/Glycine conjugated BS	0.66–1.13 [49,61,62]	0.23–0.9 [62,63,64,69,70]	0.41–1.45 [55,62]	
Healthy patients (literature):	Measured from serum		Measured from bile	
ALT/AST U/L	1.42 ± 0.02 [72]		N/A	
Bilirubin (µmol/L)	12.6 [73]		N/A	
Conjugated/Unconjugated BS	1.28–1.60 [49,74]		2.54 [64]	
Primary/Secondary BS	0.72–2.95 [49,59,66,74,75]		2.47–3.60 [64,69,70]	
Taurine/Glycine conjugated BS	0.27–2.40 [49,52,74,75]		0.26–0.30 [64,69]	

## Data Availability

Not applicable.

## Sample Availability

Samples of the compounds are not available from the authors.
